# Self-management and its association with coping styles and disease-related stigma in patients with chronic hepatitis C

**DOI:** 10.3389/fpubh.2025.1706279

**Published:** 2026-01-09

**Authors:** Jiali Wei, Yao Ge, Di Wu, Chun Wang, Guohong Ge, Dan Xu

**Affiliations:** 1 Jiangsu University, Zhenjiang, China; 2 Department of Hepatology, The Third People's Hospital of Zhenjiang, Zhenjiang, China

**Keywords:** chronic hepatitis C, coping styles, disease-related stigma, positive correlations, self-management behaviors

## Abstract

**Objective:**

To investigate the current status of self-management behaviors among patients with chronic hepatitis C (CHC) and analyze its relationship with coping styles and disease-related stigma.

**Methods:**

This study enrolled 192 CHC patients in our hospital during February 2023–July 2025. Data on general characteristics, treatment, self-management behaviors, coping styles, and disease-related stigma were collected and assessed using the Self-Management Behavior Scale for Chronic Hepatitis Patients, the Simplified Coping Style Questionnaire, and the Chronic Hepatitis C-Related Stigma Scale. These variables were compared across demographic and clinical subgroups, and Pearson correlation analysis was performed.

**Results:**

The median age of participants was 59.00 (53.00–68.00) years. The cohort consisted of 88 (45.83%) males and 104 (54.17%) females. The total self-management score was 94.14 ± 5.17. Patients scored significantly higher on positive coping (22.23 ± 3.37) than negative coping (11.44 ± 2.76, *p* < 0.05). The total stigma score was 69.01 ± 5.22. No differences in self-management, coping, or stigma were observed across age, gender, education, occupation, or marital status (all *p* > 0.05). Self-management was positively correlated with positive coping (*r* = 0.415, *p* < 0.001) and negatively correlated with negative coping (*r* = −0.354, *p* < 0.001) and disease-related stigma (*r* = −0.413, *p* < 0.001).

**Conclusion:**

Self-management among CHC patients is moderate, showing cross-sectional positive correlations with positive coping and negative correlations with both negative coping and disease-related stigma.

## 1 Introduction

Hepatitis C virus (HCV) infection is a major global public health challenge. According to the World Health Organization estimates, approximately 58 million people worldwide are living with chronic HCV infection, with over 1.5 million new cases reported annually (1). Without effective intervention, chronic hepatitis C (CHC) can progress to liver fibrosis, cirrhosis, and even hepatocellular carcinoma, threatening patient health and imposing a substantial socioeconomic burden (2–4). While direct-acting antiviral agents (DAAs) have improved cure rates and prompted goals for HCV elimination (5–7), successful outcomes depend not only on medication efficacy but also on patients’ self-management and sustained health behaviors. In the DAA era, CHC care has shifted from focusing solely on antiviral therapy to emphasizing long-term psychosocial adjustment and health behavior maintenance among patients who may live many years post-cure (8).

Self-management refers to the process through which patients with chronic illness regulate behaviors and emotions, monitor symptoms, adjust lifestyle, and engage with healthcare to promote health and well-being (9, 10). Extensive research has demonstrated that self-management levels in patients with chronic diseases are closely associated with disease control, complication prevention, and psychological well-being. For instance, in conditions such as diabetes, hypertension, and chronic obstructive pulmonary disease, high levels of self-management behaviors have been shown to significantly improve glycemic control, blood pressure levels, and overall quality of life (11–15). Similar patterns have also been observed in other infectious conditions; for example, among COVID-19 survivors with long-term complications, higher levels of spiritual health are closely linked to better quality of life (16). For CHC patients, self-management involves medication adherence, follow-up monitoring, lifestyle modifications, and coping with disease-related stigma and psychological stress (17). Unlike many chronic conditions, CHC involves a transition from chronic viral infection to sustained virological response, yet patients still face uncertainty about liver complications, reinfection risk, and social reactions to their diagnosis (18). Research on self-management in CHC, particularly regarding psychosocial factors such as coping styles and perceived stigma, remains limited. Evidence indicates that chronic hepatitis B and CHC are associated with neurocognitive impairment and neuropsychiatric symptoms, likely mediated by systemic inflammation and immune dysregulation, which may adversely affect attention, executive function, and mood (19, 20). These biological and neuropsychological burdens may compromise patients’ capacity to process health information, engage in complex decision-making, and sustain self-management behaviors.

Coping style describes cognitive and behavioral responses to illness and stress, generally categorized as active or passive (21, 22). Active coping (e.g., seeking support, rational problem-solving, and maintaining optimism) can enhance disease management and psychological adaptation. In contrast, passive coping, manifested as avoidance, denial, fantasy, or over-reliance on others, often leads to poorer treatment adherence and disease management. Previous studies have indicated that patients with chronic hepatitis B, human immunodeficiency virus (HIV) infection, or cancer who adopt active coping strategies tend to exhibit better self-management behaviors and higher quality of life, whereas passive coping may exacerbate anxiety, depression, and social isolation (23, 24). However, research specifically examining the relationship between coping styles and self-management behaviors in CHC patients remains scarce. In clinical practice, CHC patients frequently report ambivalence about disclosing their diagnosis, fear of misunderstanding by family members or employers, and persistent worries about disease progression, all of which may influence how they cope with illness-related stress (25).

Disease stigma is a significant factor affecting the psychological state and social functioning of CHC patients (26). Due to associations with transmission routes such as blood transfusion, injection drug use, and sexual contact, HCV infection is often linked to high-risk behaviors (e.g., drug use, unprotected sex), leading to discrimination and social exclusion. Stigma can lead to negative emotions, social isolation, and behaviors that undermine self-management, such as concealing their condition, avoiding medical care, or neglecting treatment. Studies have shown that strong perceptions of stigma are common among patients with hepatitis B virus (HBV) and HIV, and are closely linked to poor self-management and treatment adherence (27–29). Although HCV and HBV share similarities in transmission routes and social perceptions, research on the relationship between disease-related stigma and self-management in CHC is still limited. Importantly, even after achieving virological cure with DAAs, many CHC patients continue to experience lingering stigma, including worries about being perceived as infectious, fear of disclosure in intimate and occupational settings, and internalized shame related to presumed routes of infection. At the same time, routine patient education in busy hepatology clinics often concentrates on antiviral regimens and follow-up schedules, while providing relatively limited guidance on long-term psychosocial adjustment, disclosure decision-making, and stigma management. These CHC-specific psychosocial challenges may uniquely shape patients’ motivation, confidence, and opportunities to engage in effective self-management behaviors.

Therefore, this study aims to investigate self-management behaviors among 192 CHC patients and examine their relationships with coping styles and disease-related stigma. By focusing on a CHC cohort in the DAA era and jointly assessing self-management, coping styles, and CHC-related stigma, this study seeks to clarify how CHC-specific psychosocial challenges translate into concrete self-management patterns. The findings may provide a basis for optimizing disease management and developing targeted interventions. Drawing on the Health Belief Model and Self-Determination Theory, we hypothesized that positive (adaptive) coping would be positively associated with self-management, whereas negative (maladaptive) coping and higher levels of CHC-related stigma would be negatively associated with self-management, after accounting for basic sociodemographic and clinical characteristics.

## 2 Materials and methods

### 2.1 Patient population

**Figure 1:**
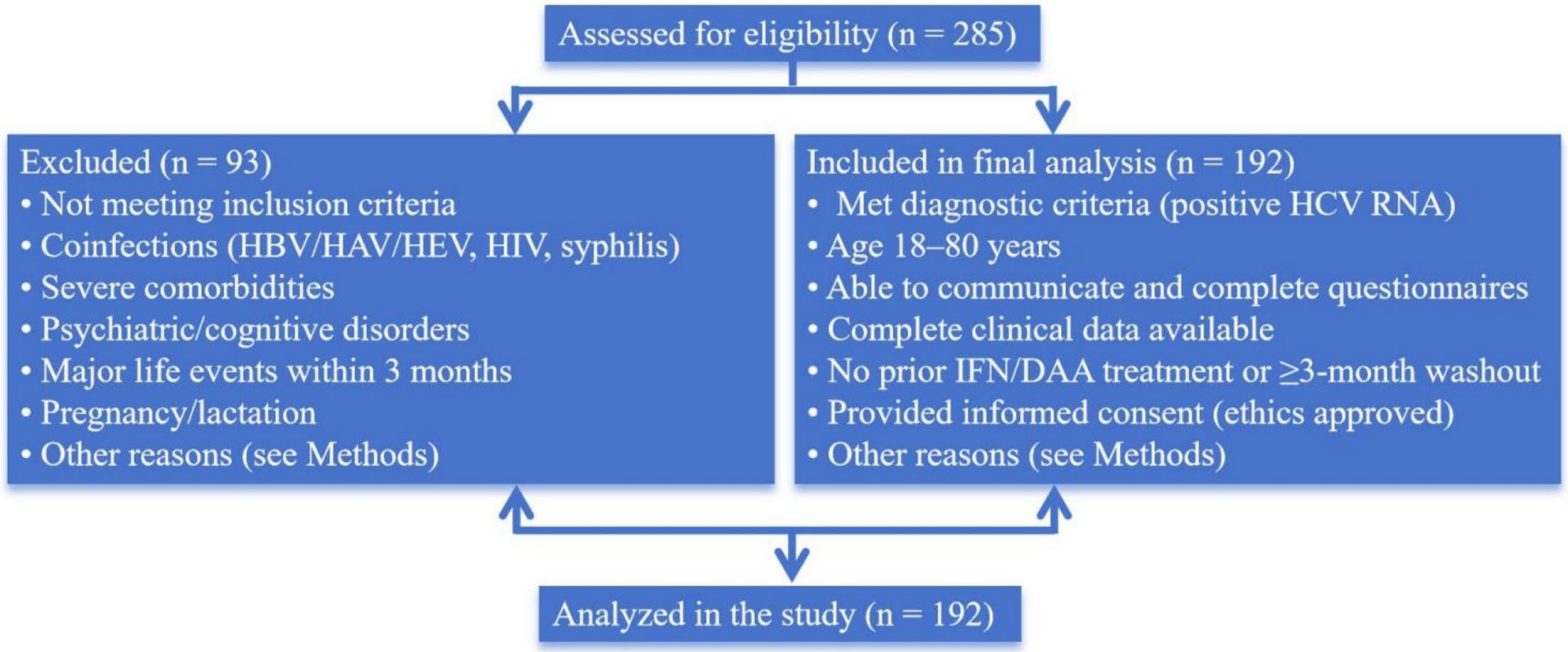
A participant flow diagram.

A total of 192 patients diagnosed with and treated for CHC in the Department of Infectious Diseases or Hepatology of The Third People’s Hospital of Zhenjiang between February 2023 and July 2025 were enrolled in this study. The inclusion criteria were as follows: (1) meeting the diagnostic criteria outlined in the *Guidelines for the Prevention and Treatment of Hepatitis C (2022 Edition)* (30) with confirmation by positive serum HCV RNA detection; (2) age between 18 and 80 years; (3) having clear consciousness and possessing basic communication skills, capable of completing questionnaires independently or with guidance from the investigator; (4) availability of complete clinical data, including key parameters such as liver function, HCV RNA viral load, and imaging examinations; (5) no prior treatment with interferon or DAAs, or a treatment-free interval of at least 3 months (to avoid the influence of therapy on self-management behaviors); and (6) approval by The Third People’s Hospital of Zhenjiang’s ethics committee and provision of informed consent by the patient. Patients were excluded from the study based on the following criteria: (1) acute or unclassified HCV infection; (2) coinfection with other viral hepatitis types including HBV, hepatitis A virus (HAV), or hepatitis E virus (HEV); (3) coinfection with HIV or syphilis, or other diseases that may contribute to perceived stigma; (4) severe dysfunction of vital organs such as the heart, brain, or kidneys, including conditions such as heart failure, uremia, or stroke; (5) diagnosed psychiatric disorders such as depression or schizophrenia, or cognitive impairments including Alzheimer’s disease or dementia, which could compromise the validity of questionnaire responses; (6) experience of major life events within the preceding 3 months, such as bereavement, divorce, or unemployment, that could significantly affect psychological status; (7) pregnancy or lactation; or (8) death during the follow-up period. Participants were enrolled using a consecutive sampling approach in the outpatient clinics of the Infectious Diseases and Hepatology departments at our hospital, ensuring that all eligible patients presenting during the study period were invited to participate. Sampling procedures were kept consistent throughout the entire recruitment period to maintain homogeneity. During the recruitment period, a total of 285 patients with confirmed CHC attended these clinics. After applying the inclusion and exclusion criteria, 192 eligible patients were consecutively enrolled. A participant flow diagram illustrating the numbers of patients screened, excluded, and included in the final analysis has been added as Figure 1. Based on an *a priori* power calculation assuming a small-to-moderate correlation (r ≈ 0.25) between self-management and key psychosocial variables, a two-sided ɑ of 0.05, and 80% power, a minimum sample size of approximately 160 participants was required; thus, the final sample of 192 patients provided adequate statistical power for the planned analyses.

### 2.2 Study methods

1. General clinical characteristics, including age, gender, educational level, occupation type, and marital status, were collected via the electronic medical record (EMR) system.
2. Clinical diagnostic and treatment data were also extracted from the EMR system, encompassing HCV RNA viral load (IU/mL), alanine aminotransferase (ALT), aspartate aminotransferase (AST), platelet count (PLT), and results from liver ultrasonography or computed tomography (CT) examinations.
3. Self-management behaviors were assessed using a hospital-developed Self-Management Behavior Scale for Chronic Hepatitis Patients. This instrument was refined through two rounds of the Delphi method, demonstrating a strong expert engagement coefficient of 0.89 and an authority coefficient of 0.86. The scale comprises 25 items distributed across five dimensions: treatment adherence (6 items), diet management (4 items), symptom monitoring (4 items), daily routine (5 items), and psychological adjustment (6 items). Responses were gauged on a 5-point Likert scale, ranging from “never” (1 point) to “always” (5 points), yielding a total score of 25–125, where higher scores indicate more effective self-management behaviors. The scale exhibited good reliability, with a Cronbach’s *α* coefficient of 0.87 and a test–retest reliability coefficient of 0.82. Although the instrument has not been directly validated against widely used international self-management scales (e.g., the Chronic Disease Self-Management Program instruments), preliminary psychometric testing-including internal consistency (Cronbach’s *α* = 0.87), test–retest reliability (0.82), and content validity (CVI = 0.91)-supports its applicability to the CHC population. The internal consistency of the Self-Management Behavior Scale for Chronic Hepatitis Patients is presented in Supplementary Table S1.
4. Coping styles were evaluated using the Simplified Coping Style Questionnaire (SCSQ), developed by Xie (31). This 20-item instrument measures two dimensions: positive coping (12 items) and negative coping (8 items). A 4-point Likert scale was employed, ranging from “never adopted” (0 point) to “regularly adopted” (3 points). Scores for the positive coping dimension range from 0 to 36, while scores for the negative coping dimension range from 0 to 24. Higher scores on a respective dimension indicate a greater propensity towards that specific coping style. The overall Cronbach’s *α* for the questionnaire was 0.90, with subscale coefficients of 0.89 for positive coping and 0.78 for negative coping, confirming its suitability for the study population.
5. Disease-related stigma was measured using a Chronic Hepatitis C-Related Stigma Scale, adapted from the Hepatitis B Virus Infection-Related Stigma Scale (HBVISS) (32). The adaptation involved modifying item wording to reflect CHC-specific context (e.g., replacing “hepatitis B” with “hepatitis C”). The scale retains 23 items across five original dimensions: received stigma (5 items), negative self-perception (4 items), perceived stigma (7 items), disease secrecy (4 items), and secondary stigma (3 items). A 5-point Likert scale was used, from “strongly disagree” (1 point) to “strongly agree” (5 points), producing a total score between 23 and 115, with higher scores indicating a greater perceived disease stigma. The adapted scale demonstrated strong psychometric properties, with an overall Cronbach’s *α* of 0.86, subscale α ranging from 0.75 to 0.83, and a content validity index (CVI) of 0.91, meeting standard measurement requirements.

### 2.3 Data collection

Prior to data collection, two researchers who had undergone standardized training conducted a unified review of the study protocol and scale administration guidelines to ensure consistency in assessment criteria. Data acquisition was carried out in a quiet, private consultation room either on the day of the patient’s outpatient visit or during their hospital stay, deliberately scheduled to avoid acute treatment phases. The collection process involved several key steps. First, the study’s purpose, content, data usage, and privacy protection measures were explained in detail to each patient. Following this, written informed consent was obtained, and a consent form was signed. The data were gathered using a combined method of one-on-one interviews and self-administered questionnaires. For patients with visual impairments, low educational level (primary school or less), or difficulties in writing, researchers read each item aloud verbatim using a standardized script and objectively recorded the answers based on the patients’ verbal responses, carefully avoiding any suggestive guidance. Upon completion, the researchers immediately checked the questionnaire for filling completeness. Any omitted or erroneously filled items were promptly confirmed and corrected with the patient to ensure an effective questionnaire return rate of ≥ 95%. Clinical data were extracted from the hospital’s EMR system, and a second researcher performed cross-verification of laboratory results and treatment records to guarantee data accuracy. All raw data were coded and entered into an EpiData 3.1 database. A double-blind entry procedure was employed for data input, followed by logical checks and consistency verification to minimize data errors.

### 2.4 Statistical analysis

Data analysis was performed using SPSS Statistics version 27.0, and graphs were generated with GraphPad Prism version 8.0.2. The normality of all continuous variables was assessed using the Shapiro–Wilk test. Quantitative data conforming to a normal distribution were presented as mean ± standard deviation ( $\bar{x}\pm s$ ), and comparisons between two groups were conducted using independent samples *t*-tests. Non-normally distributed quantitative data were expressed as median (interquartile range) [M (P25, P75)], and comparisons were performed using the Mann–Whitney *U*-test. Categorical data were summarized as frequency (percentage) [*n* (%)], and intergroup comparisons were made using the Chi-square (*χ*^2^) test. When the theoretical frequency was less than 5, the continuity-corrected *χ*^2^ test or Fisher’s exact test was applied as appropriate. For correlation analysis, the Pearson correlation coefficient was used if both variables were normally distributed; otherwise, the Spearman’s rank correlation coefficient was employed. All statistical tests were two-tailed, and a significance level of *α* = 0.05 was adopted, with a *p*-value of less than 0.05 considered statistically significant.

## 3 Results

### 3.1 General clinical characteristics and clinical data of CHC patients

Analysis of the general clinical characteristics of the 192 enrolled CHC patients revealed a median age of 59.00 (53.00, 68.00) years. The cohort comprised 88 males (45.83%) and 104 females (54.17%). Regarding educational level, 82 patients (42.71%) had a primary school level education or lower, 78 (40.63%) had completed junior to senior high school, and 32 (16.67%) had a college degree or higher. In terms of occupation, 106 patients (55.21%) were engaged in manual labor, 27 (14.07%) in intellectual labor, and 59 (30.73%) were unemployed or retired. Marital status distribution was as follows: 142 patients (73.96%) were married, 16 (8.33%) were unmarried, 22 (11.46%) were divorced, and 12 (6.25%) were widowed. Assessment of clinical parameters showed a median HCV RNA viral load of 1,340,000 (355,000, 3,855,000) IU/mL. The median ALT level was 59.50 (33.00, 126.00) U/L and the median AST level was 54.00 (33.50, 90.00) U/L. The median PLT was 122.00 (89.00 to 169.00) × 10^9^/L. Liver ultrasonography or CT imaging results indicated no abnormalities in 69 patients (35.94%), fatty liver disease in 31 (16.15%), liver cirrhosis in 34 (17.71%), and other findings in the remaining 58 patients (30.21%). The sample was predominantly older and had relatively low educational attainment, reflecting the demographic profile of CHC patients attending our tertiary hospital; therefore, caution is needed when generalizing these findings to younger or more highly educated CHC populations.

### 3.2 Self-management behaviors in CHC patients

Assessment of self-management behaviors among the 192 CHC patients revealed median scores of 25.00 (24.00–27.00) for treatment adherence, 17.00 (16.00–18.00) for diet management, 16.00 (14.00–17.00) for symptom monitoring, 16.00 (14.00–18.00) for daily routine, and 21.00 (19.00–23.00) for psychological adjustment. The total self-management score was 94.14 ± 5.17. A detailed item-by-item breakdown of responses and scores is provided in Table 1. Detailed item-by-item survey data are provided in Supplementary Table S2.

**Table 1:** Survey results of self-management behaviors in CHC patients [*n* = 192, *n* (%), score].

Item	Score
Treatment adherence	25.00 (24.00, 27.00)
Diet management	17.00 (16.00, 18.00)
Symptom monitoring	16.00 (14.00, 17.00)
Daily routine	16.00 (14.00, 18.00)
Psychological adjustment	21.00 (19.00, 23.00)
Total score	94.14 ± 5.17

### 3.3 Coping styles in CHC patients

Assessment of coping styles among the 192 CHC patients revealed a statistically significant difference (*p* < 0.05) between the two dimensions. The mean score for the positive coping dimension was 22.23 ± 3.37, which was notably higher than the mean score of 11.44 ± 2.76 for the negative coping dimension. The detailed distribution of responses for each item is presented in Table 2. Detailed item-by-item survey data are provided in Supplementary Table S3.

**Table 2:** Survey results of coping styles in CHC patients [*n* = 192, *n* (%), score].

Item	Score
Positive coping	22.23 ± 3.37
Negative coping	11.44 ± 2.76

### 3.4 Disease-related stigma in CHC patients

The assessment of disease-related stigma among the 192 CHC patients revealed median scores of 12.00 (11.00, 14.00) for received stigma, 13.00 (11.00, 14.00) for negative self-perception, 21.00 (19.00, 23.00) for perceived stigma, 16.00 (14.00, 17.00) for disease secrecy, and 7.00 (6.00, 9.00) for secondary stigma. The total stigma score was 69.01 ± 5.22 (Table 3). Detailed item-by-item survey data are provided in Supplementary Table S4.

**Table 3:** Survey results of disease-related stigma in CHC patients [*n* = 192, *n* (%), score].

Item	Score
Received stigma	12.00 (11.00, 14.00)
Negative self-perception	13.00 (11.00, 14.00)
Perceived stigma	21.00 (19.00, 23.00)
Disease secrecy	16.00 (14.00, 17.00)
Total score	69.01 ± 5.22

### 3.5 Differences in self-management behaviors, coping styles, and disease-related stigma according to clinical characteristics

Comparisons of self-management behaviors, coping styles, and disease-related stigma across subgroups defined by age, gender, educational level, occupation type, and marital status revealed no statistically significant differences (*p* > 0.05 for all comparisons). The detailed results are presented in Table 4.

**Table 4:** Differences in self-management behaviors, coping styles, and disease-related stigma by clinical characteristics.

Clinical characteristic	*n*	Self-management behaviors	Positive coping	Negative coping	Disease-related stigma
Age					
31 ~ 50 years	33	94.82 ± 5.70	22.24 ± 3.65	11.61 ± 3.12	67.70 ± 5.90
51 ~ 80 years	159	94.00 ± 5.06	22.23 ± 3.32	11.41 ± 2.69	69.28 ± 5.04
Gender					
Male	88	94.31 ± 5.54	22.20 ± 3.19	12.00 (10.00, 13.50)	68.74 ± 5.31
Female	104	94.00 ± 4.86	22.26 ± 3.53	11.00 (9.00, 13.00)	69.24 ± 5.15
Education					
Primary or below	82	93.82 ± 5.33	22.29 ± 3.42	11.00 (9.00, 13.00)	69.04 ± 4.94
Junior–Senior High	78	94.01 ± 4.95	22.28 ± 3.16	12.00 (10.00, 14.00)	69.38 ± 5.50
College or above	32	95.28 ± 5.29	21.97 ± 3.82	12.00 (10.50, 13.50)	68.03 ± 5.25
Occupation					
Manual labor	106	94.15 ± 4.78	22.17 ± 3.41	11.60 ± 2.73	69.11 ± 4.91
Intellectual labor	27	94.41 ± 4.55	22.59 ± 2.72	11.19 ± 2.80	69.37 ± 5.69
Unemployed/Retired	59	94.00 ± 6.11	22.19 ± 3.61	11.27 ± 2.83	68.66 ± 5.59
Marital status					
Married	142	94.15 ± 5.12	22.10 ± 3.42	11.66 ± 2.85	69.14 ± 5.35
Unmarried	16	95.00 ± 5.23	22.75 ± 2.77	10.88 ± 2.99	68.69 ± 6.34
Divorced	22	93.68 ± 5.30	23.55 ± 2.81	10.95 ± 2.48	69.36 ± 4.02
Widowed	12	93.67 ± 5.94	20.75 ± 3.93	10.50 ± 1.38	67.25 ± 3.96

### 3.6 Correlations of self-management behaviors with coping styles and disease-related stigma in CHC patients

**Figure 2:**
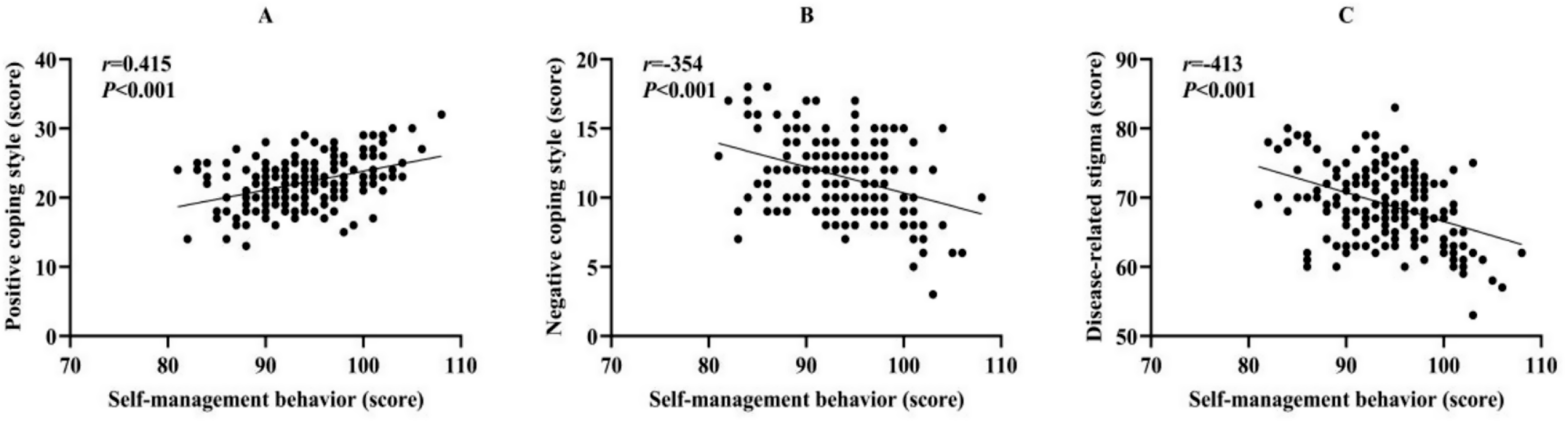
Correlations between self-management behaviors and coping styles/disease-related stigma in CHC patients. **(A)** Scatter plot of the correlation between self-management behaviors and positive coping styles; **(B)** Scatter plot of the correlation between self-management behaviors and negative coping styles; **(C)** Scatter plot of the correlation between self-management behaviors and disease-related stigma.

Pearson correlation analysis revealed that self-management behaviors demonstrated a moderate positive correlation with positive coping styles (*r* = 0.415, 95%CI: 0.291 ~ 0.526, *p* < 0.001). Conversely, a weak negative correlation was observed between self-management behaviors and negative coping styles (*r* = −0.354, 95%CI: −0.472 ~ −0.224, *p* < 0.001). Furthermore, self-management behaviors exhibited a moderate negative correlation with disease-related stigma (*r* = −0.413, 95%CI: −0.524 ~ −0.288, *p* < 0.001). These correlations are visually represented in Figure 2.

## 4 Discussion

This study indicates that the overall self-management proficiency of CHC patients is at a moderate level. Self-management was positively correlated with positive coping, and negatively correlated with both negative coping and disease-related stigma. From the perspective of contemporary psychosocial and behavioral theories, these patterns suggest that CHC self-management is not a set of isolated behaviors, but the product of patients’ illness perceptions, motivation, and social context. In particular, key elements of the Health Belief Model (e.g., perceived severity, perceived benefits and barriers, and self-efficacy) and Self-Determination Theory (e.g., autonomy, competence, and relatedness) provide a useful framework for interpreting how coping styles and stigma influence self-management in this population. These results provide insights into the key psychosocial factors associated with self-management in CHC patients, offering multi-faceted implications for clinical intervention. From a biological perspective, these findings should also be interpreted against the backdrop of emerging data that chronic viral hepatitis is linked to neurocognitive impairment and neuropsychiatric symptoms via systemic inflammation and immune dysregulation (33), suggesting that some CHC patients may face double burdens of hepatic disease and subtle brain dysfunction that jointly constrain psychosocial adjustment and self-management capacity. Similarly, recent COVID-19 research has highlighted that immune-inflammatory perturbations-such as vitamin D-related alterations in platelet indices, genetic variation in platelet antigens and complement pathway components, and post-chemotherapy declines in vaccine-induced IgG-may shape vulnerability to infection and downstream clinical outcomes (34–36).

In the present study, the total self-management score among CHC patients was 94.14 ± 5.17, indicating acceptable yet suboptimal self-management proficiency with considerable room for improvement. Analysis across dimensions revealed relatively higher scores in treatment adherence and diet management, whereas deficiencies were observed in daily routine management and psychological adjustment. Consistent with studies involving patients with other chronic conditions such as diabetes and hypertension (37, 38), this study underscores the critical importance of self-management in the long-term control of chronic diseases. From the Health Belief Model perspective, higher scores in medication and diet management may reflect patients’ recognition of their benefits. In contrast, lower scores in daily routine and psychological adjustment could indicate that these areas are not viewed as critical health behaviors, resulting in weaker perceived benefits and lower self-efficacy. Moreover, from a Self-Determination Theory perspective, limited opportunities to develop competence (e.g., insufficient skills training in stress management and sleep hygiene) and autonomy (e.g., prescriptive rather than collaborative communication in clinic visits) may help explain why CHC patients find it more difficult to sustain self-regulation in these less “visible” areas of self-management. These gaps may be attributed to public misconceptions regarding HCV infection, patients’ own cognitive limitations, and insufficient patient-provider communication (39). Consequently, healthcare professionals should emphasize not only treatment adherence but also integrated management of lifestyle and psychological aspects during interventions.

In addition, our study identified a significant positive correlation between self-management behaviors and positive coping styles. Positive coping facilitates patients’ active acquisition of disease-related information, seeking of social support, and adoption of effective health behaviors, a finding consistent with previous research on patients with HBV, HIV, and cancer (40). Conversely, a negative correlation was observed between self-management and negative coping styles, suggesting that patients who adopt avoidance- or dependency-based strategies tend to demonstrate poorer performance in medication adherence, follow-up monitoring, and lifestyle modifications. Psychological theories posit that positive coping enhances an individual’s sense of control over their disease, whereas negative coping reinforces feelings of helplessness and dependency, thereby compromising self-management efficacy (41). Self-determination theory offers another lens. Positive coping supports basic psychological needs of autonomy (through active decision-making and problem-solving) and competence (through effective symptom management). This support fosters more internalized, self-determined motivation for self-management. In contrast, avoidance-based or overly dependent coping may thwart autonomy and competence, leading to more externally driven or unmotivated regulation, and consequently weaker adherence to self-management behaviors. From the standpoint of the Health Belief Model, positive coping may also promote more accurate risk appraisal and stronger perceived benefits, whereas negative coping may amplify perceived barriers and minimize cues to action, further undermining sustained behavior change. Thus, one of the key strategies for improving self-management in CHC patients may involve fostering positive coping patterns through psychological counseling, peer education, and cognitive behavioral therapy to promote adaptive thinking and behavioral patterns.

Moreover, this study revealed that the average disease-related stigma score among CHC patients was 69.01 ± 5.22, indicating a moderately high level of perceived stigma, which was negatively correlated with self-management behaviors. The presence of stigma may lead patients to avoid seeking medical care, reduce social interactions, and experience negative emotions, thereby directly undermining self-management behaviors (42). For instance, concerns about being identified may cause patients to skip follow-up visits, or shame may prevent open communication with healthcare providers. Within the Health Belief Model, stigma can be conceptualized as a powerful perceived barrier that discourages disclosure, clinic attendance, and engagement in recommended behaviors, even when patients recognize the severity of CHC and the benefits of treatment. Self-Determination Theory further suggests that stigma may erode patients’ sense of relatedness (e.g., feeling rejected or misunderstood by others) and competence (e.g., believing oneself to be “incurable” or “contaminated”), thereby weakening internal motivation to care for one’s health. In the DAA era, lingering stigma after virological cure may thus partially explain why some patients do not fully translate medical “cure” into sustained, autonomous self-management in daily life. In clinical practice, interventions to reduce stigma should include: strengthening public health education to correct misconceptions about HCV transmission; fostering a non-discriminatory healthcare environment to alleviate psychological burden; and providing psychological support and social resources for patients and their families to help rebuild self-identity. It is also important to interpret these patterns within the specific cultural context of Chinese CHC patients. In our single-institution sample, traditional norms surrounding “face,” family responsibility, and gender roles may shape how patients disclose their diagnosis, seek support, and cope with stigma. For example, some patients may prioritize not burdening family members or avoid discussing illness openly, which could foster more avoidant coping and stronger internalized stigma despite good treatment adherence. These contextual factors may partly account for the observed associations between stigma, coping, and self-management and should be considered when designing culturally sensitive interventions. Although we did not observe significant sex differences in self-management, coping styles, or CHC-related stigma in the present sample, prior research suggests that gender can modulate health-related behaviors and psychosocial adjustment (43–45). The absence of sex effects in our data may reflect sample characteristics, cultural norms regarding gender roles, or the limited sensitivity of our measures to capture gender-specific experiences. Future studies should therefore examine sex and gender as potential moderators or mediators in the pathways linking stigma, coping, and self-management, for example by testing whether the strength of these associations differs between men and women.

Taken together, these findings suggest that theory-informed interventions may be particularly beneficial for CHC patients. From a Health Belief Model standpoint, interventions should aim to enhance perceived benefits and self-efficacy (e.g., clear information about how specific behaviors reduce long-term liver risks), while reducing perceived barriers associated with stigma and practical constraints (e.g., concerns about disclosure at work or in families). From a Self-Determination Theory perspective, effective programs should support patients’ autonomy (e.g., shared decision-making, respecting preferences regarding disclosure), competence (e.g., skills training in symptom monitoring, stress management, and communication), and relatedness (e.g., peer support, non-judgmental clinical interactions) to foster more internalized motivation for sustained self-management. Based on these theoretical and empirical insights, structured CHC self-management programs that integrate stigma-reduction components and coping-skills training should be developed and evaluated. Improving self-management among CHC patients may be achieved through the following integrated approaches: (1) health education interventions utilizing individualized instruction and online-offline campaigns to enhance awareness of the disease and self-management importance; (2) psychological interventions such as counseling, group therapy, and cognitive behavioral therapy to encourage positive coping and reduce negative thinking; (3) social support enhancements involving family, community, and medical institutions to reduce discrimination and ensure equitable healthcare and employment opportunities; and (4) multidisciplinary management models that integrate hepatology, psychology, and nursing teams to establish patient-centered care combining medical treatment with psychosocial support. Incorporating biological and neuropsychological perspectives into such models—by routinely screening for cognitive/mood disturbances and addressing inflammation-related neuropsychiatric symptoms-would further strengthen the argument that effective CHC management must simultaneously target hepatic, neurocognitive, and psychosocial dimensions.

Nevertheless, this study has several limitations. First, as a single-center cross-sectional survey, it cannot establish causal relationships, and future multi-center longitudinal studies are warranted. In addition, our single-center sample was skewed toward older patients with relatively low educational attainment, which may introduce selection bias and limit the generalizability of the findings to other settings and demographic groups. Second, despite efforts to minimize bias, all key variables were measured using self-report questionnaires at a single time point, so the data are subject to social desirability and recall biases, and common method bias or shared method variance may have inflated the observed associations between psychosocial factors and self-management; future studies should combine self-report measures with objective clinical indicators or multi-informant assessments. Third, the study did not thoroughly examine differences in self-management across disease stages (e.g., cirrhotic vs. non-cirrhotic patients), so future research could stratify populations accordingly. Moreover, although information on DAA treatment status and viral load was collected, the study was not powered for extensive subgroup or sensitivity analyses (e.g., treated vs. untreated, high vs. low viral load), and we did not conduct these comparisons; larger samples will be needed to clarify whether self-management patterns differ across specific clinical subgroups. Fourth, although preliminary psychometric testing of the Self-Management Behavior Scale for Chronic Hepatitis Patients demonstrated acceptable internal consistency, test–retest reliability, and content validity, confirmatory factor analysis was not conducted, and model-fit indices (e.g., CFI, TLI, RMSEA) are therefore unavailable; future studies should further validate the factor structure of this instrument in diverse CHC populations. In addition, although the adapted CHC stigma scale showed good internal consistency and content validity in our sample, it has not yet been fully validated against established HCV-specific stigma instruments, which may limit the comparability of our findings with those from studies using widely adopted stigma measures. Fifth, our analytic strategy was primarily correlation-based and did not include multivariable or hierarchical regression models to identify independent predictors of self-management, so residual confounding by unmeasured or weakly associated sociodemographic and clinical factors cannot be entirely ruled out. In particular, we did not systematically assess or adjust for depressive/anxiety symptoms, detailed socioeconomic indicators, substance use disorders, or perceived social support, even though these variables are known to be related to both coping and stigma, and future studies should incorporate these factors in more comprehensive multivariable or path-analytic models. Finally, although we interpreted our findings in light of the Health Belief Model and Self-Determination Theory, we did not directly assess specific theoretical constructs (e.g., perceived barriers, autonomous vs. controlled motivation), and future studies should incorporate validated theory-based measures to more precisely test these mechanisms and inform targeted interventions. Additionally, employing qualitative methods could provide deeper insights into the psychological mechanisms linking stigma and self-management from the patients’ subjective perspectives.

## 5 Conclusion

In conclusion, this study demonstrates that self-management among CHC patients is generally moderate and is significantly associated with coping styles and disease-related stigma. In this cross-sectional sample, positive coping strategies were associated with higher self-management scores, whereas negative coping and higher levels of stigma were associated with lower self-management scores. Future clinical initiatives should implement comprehensive interventions that address not only disease treatment but also psychological support and socio-environmental improvements, aiming to holistically enhance health outcomes and quality of life for CHC patients.

## Data Availability

The original contributions presented in the study are included in the article/supplementary material, further inquiries can be directed to the corresponding author.
