# Supplementary material for: Self-management and its association with coping styles and disease-related stigma in patients with chronic hepatitis C
Source: Front Public Health. 2026 Jan 9;13:1706279. doi: 10.3389/fpubh.2025.1706279 (PMC12827648; doi:10.3389/fpubh.2025.1706279)
Supplement: Supplementary file 1 [file Table_1.DOCX]

Supplementary Table S1 Internal Consistency of the Self-Management Behavior Scale for Chronic Hepatitis Patients

| Subscale | Number of Items | Cronbach’s α |
| --- | --- | --- |
| Treatment adherence | 6 | 0.78 |
| Diet management | 4 | 0.72 |
| Symptom monitoring | 4 | 0.7 |
| Daily routine | 5 | 0.68 |
| Psychological adjustment | 6 | 0.75 |
| Total scale | 25 | 0.87 |
| Scale-level content validity (CVI) | - | 0.91 |

Note: Cronbach’s α coefficients were calculated to assess internal consistency at both the subscale and total scale levels. Subscale α values ranged from 0.68 to 0.78, indicating acceptable to good reliability, while the overall scale demonstrated excellent internal consistency (α = 0.87). In addition, the scale-level content validity index (CVI) was 0.91 based on expert ratings, indicating good content validity. The results support the reliability of the instrument for evaluating self-management behaviors among patients with chronic hepatitis C.
