# Supplementary material for: Self-management and its association with coping styles and disease-related stigma in patients with chronic hepatitis C
Source: Front Public Health. 2026 Jan 9;13:1706279. doi: 10.3389/fpubh.2025.1706279 (PMC12827648; doi:10.3389/fpubh.2025.1706279)
Supplement: Supplementary file 2 [file Table_2.DOCX]

**Supplementary Table S2 Survey results of self-management behaviors in CHC patients [n = 192, n (%), score]**

| **Item** | **Never** | **Rarely** | **Sometimes** | **Often** | **Always** | **Score** |
| --- | --- | --- | --- | --- | --- | --- |
| **Treatment adherence** |  |  |  |  |  | 25.00 (24.00, 27.00) |
| 1. I take my hepatitis medications exactly as prescribed (on time and in the correct dose). | 0 (0.00) | 0 (0.00) | 0 (0.00) | 49 (25.52) | 143 (74.48) | 5.00 (4.00, 5.00) |
| 2. I attend follow-up appointments as required by my doctor. | 0 (0.00) | 4 (2.08) | 18 (9.38) | 78 (40.63) | 92 (47.92) | 4.00 (4.00, 5.00) |
| 3. I avoid medications that can damage liver function. | 0 (0.00) | 0 (0.00) | 2 (1.04) | 83 (43.23) | 107 (55.73) | 5.00 (4.00, 5.00) |
| 4. I maintain good communication with healthcare staff. | 9 (4.69) | 22 (11.46) | 46 (23.96) | 63 (32.81) | 52 (27.08) | 4.00 (3.00, 5.00) |
| 5. I consult healthcare staff about hepatitis-related issues. | 6 (3.13) | 15 (7.81) | 42 (21.88) | 68 (35.42) | 61 (31.77) | 4.00 (3.00, 5.00) |
| 6. I work with healthcare staff to develop a disease management plan. | 4 (2.08) | 10 (5.21) | 38 (19.79) | 76 (39.58) | 64 (33.33) | 4.00 (3.00, 5.00) |
| **Diet management** |  |  |  |  |  | 17.00 (16.00, 18.00) |
| 7. I limit my intake of high-fat, high-cholesterol, or irritating foods. | 2 (1.04) | 8 (4.17) | 33 (17.19) | 88 (45.83) | 61 (31.77) | 4.00 (4.00, 5.00) |
| 8. I ensure I consume enough protein daily. | 0 (0.00) | 4 (2.08) | 26 (13.54) | 94 (48.96) | 68 (35.42) | 4.00 (4.00, 5.00) |
| 9. I eat an adequate amount of fresh vegetables and fruit daily. | 0 (0.00) | 3 (1.56) | 22 (11.46) | 97 (50.52) | 70 (36.46) | 4.00 (4.00, 5.00) |
| 10. I completely abstain from alcohol. | 0 (0.00) | 0 (0.00) | 3 (1.56) | 67 (34.90) | 122 (63.54) | 5.00 (4.00, 5.00) |
| **Symptom monitoring** |  |  |  |  |  | 16.00 (14.00, 17.00) |
| **11. I proactively monitor myself for hepatitis-related symptoms.** | 7 (3.65) | 23 (11.98) | 48 (25.00) | 66 (34.38) | 48 (25.00) | 4.00 (3.00, 4.50) |
| **12. I seek immediate medical attention if symptoms such as fever, fatigue, liver discomfort, or jaundice worsen.** | 0 (0.00) | 2 (1.04) | 12 (6.25) | 72 (37.50) | 106 (55.21) | 5.00 (4.00, 5.00) |
| **13. I use methods like warm compresses or rest to relieve liver discomfort or fatigue.** | 11 (5.73) | 26 (13.54) | 53 (27.60) | 62 (32.29) | 40 (20.83) | 4.00 (3.00, 4.00) |
| **14. I keep complete medical records and test reports for use during follow-up visits.** | 0 (0.00) | 17 (8.85) | 38 (19.79) | 76 (39.58) | 61 (31.77) | 4.00 (3.00, 5.00) |
| **Daily routine** |  |  |  |  |  | 16.00 (14.00, 18.00) |
| **15. I balance exercise and rest appropriately based on my symptoms and fatigue levels.** | 5 (2.60) | 18 (9.38) | 51 (26.56) | 72 (37.50) | 46 (23.96) | 4.00 (3.00, 4.00) |
| **16. I ensure I get 7-8 hours of sleep per night.** | 13 (6.77) | 33 (17.19) | 58 (30.21) | 54 (28.13) | 34 (17.71) | 3.00 (3.00, 4.00) |
| **17. I maintain a regular daily schedule.** | 14 (7.29) | 38 (19.79) | 63 (32.81) | 52 (27.08) | 25 (13.02) | 3.00 (2.00, 4.00) |
| **18. I exchange disease management experiences with other hepatitis patients.** | 51 (26.56) | 43 (22.40) | 42 (21.88) | 30 (15.63) | 26 (13.54) | 3.00 (1.00, 4.00) |
| **19. I learn about hepatitis through books, the internet, or lectures.** | 24 (12.50) | 48 (25.00) | 54 (28.13) | 43 (22.40) | 23 (11.98) | 3.00 (2.00, 4.00) |
| **Psychological adjustment** |  |  |  |  |  | 21.00 (19.00, 23.00) |
| **20. I maintain an optimistic outlook towards my hepatitis.** | 21 (10.94) | 46 (23.96) | 58 (30.21) | 43 (22.40) | 24 (12.50) | 3.00 (2.00, 4.00) |
| **21. I am confident in my ability to manage my hepatitis.** | 3 (1.56) | 27 (14.06) | 31 (16.15) | 64 (33.33) | 67 (34.90) | 4.00 (3.00, 5.00) |
| **22. I can self-regulate when experiencing negative emotions.** | 9 (4.69) | 26 (13.54) | 59 (30.73) | 59 (30.73) | 39 (20.31) | 4.00 (3.00, 4.00) |
| **23. I proactively cope with the social stigma associated with hepatitis.** | 26 (13.54) | 43 (22.40) | 55 (28.65) | 43 (22.40) | 25 (13.02) | 3.00 (2.00, 4.00) |
| **24. I maintain normal social activities.** | 0 (0.00) | 6 (3.13) | 19 (9.90) | 66 (34.38) | 101 (52.60) | 5.00 (4.00, 5.00) |
| **25. I ask others for help when facing difficulties in disease management.** | 27 (14.06) | 50 (26.04) | 59 (30.73) | 36 (18.75) | 20 (10.42) | 3.00 (2.00, 4.00) |
| **Total score** |  |  |  |  |  | 94.14 ± 5.17 |
