# Supplementary material for: Self-management and its association with coping styles and disease-related stigma in patients with chronic hepatitis C
Source: Front Public Health. 2026 Jan 9;13:1706279. doi: 10.3389/fpubh.2025.1706279 (PMC12827648; doi:10.3389/fpubh.2025.1706279)
Supplement: Supplementary file 3 [file Table_3.DOCX]

**Supplementary Table S3 Survey results of coping styles in CHC patients [n = 192, n (%), score]**

| **Item** | **Not adopted** | **Occasionally adopted** | **Sometimes adopted** | **Regularly adopted** | **Score** |
| --- | --- | --- | --- | --- | --- |
| **Positive coping** |  |  |  |  | 22.23 ± 3.37 |
| 1. Seeking relief through work, study, or other activities. | 14 (7.29) | 31 (16.15) | 57 (29.69) | 90 (46.88) | 2.00 (2.00, 3.00) |
| 2. Talking to others to share inner worries. | 33 (17.19) | 47 (24.48) | 63 (32.81) | 49 (25.52) | 2.00 (1.00, 3.00) |
| 3. Trying to see the positive aspects of things. | 11 (5.73) | 24 (12.50) | 61 (31.77) | 96 (50.00) | 2.50 (2.00, 3.00) |
| 4. Changing my perspective and rediscovering what is important in life. | 19 (9.90) | 43 (22.40) | 64 (33.33) | 66 (34.38) | 2.00 (1.00, 3.00) |
| 5. Not taking problems too seriously. | 17 (8.85) | 41 (21.35) | 67 (34.90) | 67 (34.90) | 2.00 (1.00, 3.00) |
| 6. Standing my ground and fighting for what I want. | 26 (13.54) | 47 (24.48) | 64 (33.33) | 55 (28.65) | 2.00 (1.00, 3.00) |
| 7. Finding several different ways to solve problems. | 23 (11.98) | 39 (20.31) | 71 (36.98) | 59 (30.73) | 2.00 (1.00, 3.00) |
| 8. Seeking advice from relatives, friends, or classmates. | 31 (16.15) | 44 (22.92) | 63 (32.81) | 54 (28.13) | 2.00 (1.00, 3.00) |
| 9. Changing some of my previous approaches or addressing personal issues. | 13 (6.77) | 34 (17.71) | 69 (35.94) | 76 (39.58) | 2.00 (2.00, 3.00) |
| 10. Learning from how others have handled similar difficult situations. | 59 (30.73) | 55 (28.65) | 51 (26.56) | 27 (14.06) | 1.00 (0.00, 2.00) |
| 11. Seeking hobbies and actively participating in recreational activities. | 37 (19.27) | 53 (27.60) | 54 (28.13) | 48 (25.00) | 2.00 (1.00, 2.50) |
| 12. Trying to restrain my disappointment, regret, sadness, and anger. | 19 (9.90) | 37 (19.27) | 63 (32.81) | 73 (38.02) | 2.00 (1.00, 3.00) |
| **Negative coping** |  |  |  |  | 11.44 ± 2.76 |
| **13. Trying to rest or take a vacation to temporarily set aside problems/worries.** | 44 (22.92) | 63 (32.81) | 53 (27.60) | 32 (16.67) | 1.00 (1.00, 2.00) |
| **14. Using smoking, drinking, medication, or eating to relieve worries.** | 121 (63.02) | 39 (20.31) | 19 (9.90) | 13 (6.77) | 0.00 (0.00, 1.00) |
| **15. Believing time will change the situation and the only thing to do is wait.** | 39 (20.31) | 61 (31.77) | 53 (27.60) | 39 (20.31) | 1.00 (1.00, 2.00) |
| 16. Trying to forget the whole thing. | 33 (17.19) | 51 (26.56) | 67 (34.90) | 41 (21.35) | 2.00 (1.00, 2.00) |
| **17. Relying on others to solve problems.** | 71 (36.98) | 50 (26.04) | 47 (24.48) | 24 (12.50) | 1.00 (0.00, 2.00) |
| **18. Accepting reality because there is no other way.** | 17 (8.85) | 33 (17.19) | 59 (30.73) | 83 (43.23) | 2.00 (1.00, 3.00) |
| **19. Fantasizing that a miracle might change the situation.** | 73 (38.02) | 54 (28.13) | 46 (23.96) | 19 (9.90) | 1.00 (0.00, 2.00) |
| **20. Comforting myself.** | 15 (7.81) | 31 (16.15) | 64 (33.33) | 82 (42.71) | 2.00 (2.00, 3.00) |
