# Supplementary material for: Self-management and its association with coping styles and disease-related stigma in patients with chronic hepatitis C
Source: Front Public Health. 2026 Jan 9;13:1706279. doi: 10.3389/fpubh.2025.1706279 (PMC12827648; doi:10.3389/fpubh.2025.1706279)
Supplement: Supplementary file 4 [file Table_4.DOCX]

**Supplementary Table S4 Survey results of disease-related stigma in CHC patients [n = 192, n (%), score]**

| **Item** | **Strongly disagree** | **Disagree** | **Neutral** | **Agree** | **Strongly agree** | **Score** |
| --- | --- | --- | --- | --- | --- | --- |
| **Received stigma** |  |  |  |  |  | 12.00 (11.00, 14.00) |
| 1. I have experienced unfair treatment (e.g., rejection for jobs, dates, marriage) due to my HCV infection. | 47 (24.48) | 63 (32.81) | 41 (21.35) | 31 (16.15) | 10 (5.21) | 2.00 (2.00, 3.00) |
| 2. My family is reluctant to talk to others about the HCV infection for fear of rejection. | 33 (17.19) | 57 (29.69) | 54 (28.13) | 37 (19.27) | 11 (5.73) | 3.00 (2.00, 3.50) |
| 3. I have been pushed aside by friends because of my HCV infection. | 51 (26.56) | 64 (33.33) | 44 (22.92) | 24 (12.50) | 9 (4.69) | 2.00 (1.00, 3.00) |
| 4. I have been denied care or contact with children because of my HCV infection. | 67 (34.90) | 59 (30.73) | 41 (21.35) | 17 (8.85) | 8 (4.17) | 2.00 (1.00, 3.00) |
| 5. I feel I am treated differently because of my HCV infection. | 24 (12.50) | 43 (22.40) | 61 (31.77) | 49 (25.52) | 15 (7.81) | 3.00 (2.00, 4.00) |
| **Negative self-perception** |  |  |  |  |  | 13.00 (11.00, 14.00) |
| 6. Since being infected with HCV, I feel unfortunate and that it is unfair. | 17 (8.85) | 31 (16.15) | 49 (25.52) | 67 (34.90) | 28 (14.58) | 3.00 (2.50, 4.00) |
| 7. Since being infected with HCV, I feel like a burden to my family/society. | 23 (11.98) | 44 (22.92) | 53 (27.60) | 54 (28.13) | 18 (9.38) | 3.00 (2.00, 4.00) |
| 8. People's attitudes towards those with HCV make me feel worse about myself. | 19 (9.90) | 37 (19.27) | 63 (32.81) | 51 (26.56) | 22 (11.46) | 3.00 (2.00, 4.00) |
| 9. I worry about being disliked and avoided, so I do not initiate interactions with others. | 14 (7.29) | 33 (17.19) | 47 (24.48) | 71 (36.98) | 27 (14.06) | 4.00 (3.00, 4.00) |
| **Perceived Stigma** |  |  |  |  |  | 21.00 (19.00, 23.00) |
| 10. Because of my HCV infection, I feel that people talk about me behind my back. | 21 (10.94) | 39 (20.31) | 54 (28.13) | 59 (30.73) | 19 (9.90) | 3.00 (2.00, 4.00) |
| 11. Because of my HCV infection, I feel that some people are uncomfortable around me. | 27 (14.06) | 44 (22.92) | 57 (29.69) | 49 (25.52) | 15 (7.81) | 3.00 (2.00, 4.00) |
| 12. Because of my HCV infection, I feel embarrassed by what people think of me. | 19 (9.90) | 41 (21.35) | 61 (31.77) | 53 (27.60) | 18 (9.38) | 3.00 (2.00, 4.00) |
| 13. I believe others treat people with HCV infection differently. | 11 (5.73) | 29 (15.10) | 53 (27.60) | 73 (38.02) | 26 (13.54) | 4.00 (3.00, 4.00) |
| 14. I believe others do not want to be friends with people who have HCV infection. | 17 (8.85) | 47 (24.48) | 59 (30.73) | 51 (26.56) | 18 (9.38) | 3.00 (2.00, 4.00) |
| 15. I believe hepatitis C prevents infected people from enjoying the same rights as ordinary people. | 24 (12.50) | 51 (26.56) | 57 (29.69) | 44 (22.92) | 16 (8.33) | 3.00 (2.00, 4.00) |
| 16. I believe hepatitis C causes infected people to lose their jobs. | 33 (17.19) | 59 (30.73) | 49 (25.52) | 41 (21.35) | 10 (5.21) | 3.00 (2.00, 4.00) |
| **Disease secrecy** |  |  |  |  |  | 16.00 (14.00, 17.00) |
| 17. I do not want to tell others that I am infected with hepatitis C. | 8 (4.17) | 13 (6.77) | 31 (16.15) | 74 (38.54) | 66 (34.38) | 4.00 (3.00, 5.00) |
| 18. I will only tell people I trust that I am infected with hepatitis C. | 6 (3.13) | 11 (5.73) | 29 (15.10) | 81 (42.19) | 65 (33.85) | 4.00 (4.00, 5.00) |
| 19. I hope those who know about my HCV infection will keep it confidential from others. | 7 (3.65) | 14 (7.29) | 33 (17.19) | 73 (38.02) | 65 (33.85) | 4.00 (3.00, 5.00) |
| 20. I am afraid that going to the hospital will let people know I am infected with HCV. | 13 (6.77) | 24 (12.50) | 41 (21.35) | 67 (34.90) | 47 (24.48) | 4.00 (3.00, 5.00) |
| **Secondary stigma** |  |  |  |  |  | 7.00 (6.00, 9.00) |
| 21. Because of my HCV infection, my relatives and friends visit me less often than before. | 47 (24.48) | 61 (31.77) | 44 (22.92) | 31 (16.15) | 9 (4.69) | 2.00 (2.00, 3.00) |
| 22. Because of my HCV infection, my family feels ashamed. | 39 (20.31) | 63 (32.81) | 51 (26.56) | 29 (15.10) | 10 (5.21) | 2.00 (2.00, 3.00) |
| 23. Because of my HCV infection, my family experiences rejection from others. | 51 (26.56) | 67 (34.90) | 41 (21.35) | 24 (12.50) | 9 (4.69) | 2.00 (1.00, 3.00) |
| **Total score** |  |  |  |  |  | 69.01 ± 5.22 |
